# COVID-19 in a Correctional Facility Employee Following Multiple Brief Exposures to Persons with COVID-19 — Vermont, July–August 2020

**DOI:** 10.15585/mmwr.mm6943e1

**Published:** 2020-10-30

**Authors:** Julia C. Pringle, Jillian Leikauskas, Sue Ransom-Kelley, Benjamin Webster, Samuel Santos, Heidi Fox, Shannon Marcoux, Patsy Kelso, Natalie Kwit

**Affiliations:** ^1^Epidemic Intelligence Service, CDC; ^2^Vermont Department of Health; ^3^Vermont Department of Corrections.

On August 11, 2020, a confirmed case of coronavirus disease 2019 (COVID-19) in a male correctional facility employee (correctional officer) aged 20 years was reported to the Vermont Department of Health (VDH). On July 28, the correctional officer had multiple brief encounters with six incarcerated or detained persons (IDPs)* while their SARS-CoV-2 test results were pending. The six asymptomatic IDPs arrived from an out-of-state correctional facility on July 28 and were housed in a quarantine unit. In accordance with Vermont Department of Corrections (VDOC) policy for state prisons, nasopharyngeal swabs were collected from the six IDPs on their arrival date and tested for SARS-CoV-2, the virus that causes COVID-19, at the Vermont Department of Health Laboratory, using real-time reverse transcription–polymerase chain reaction (RT-PCR). On July 29, all six IDPs received positive test results. VDH and VDOC conducted a contact tracing investigation^†^and used video surveillance footage to determine that the correctional officer did not meet VDH’s definition of close contact (i.e., being within 6 feet of infectious persons for ≥15 consecutive minutes)^§^^,^^¶^; therefore, he continued to work. At the end of his shift on August 4, he experienced loss of smell and taste, myalgia, runny nose, cough, shortness of breath, headache, loss of appetite, and gastrointestinal symptoms; beginning August 5, he stayed home from work. An August 5 nasopharyngeal specimen tested for SARS-CoV-2 by real-time RT-PCR at a commercial laboratory was reported as positive on August 11; the correctional officer identified two contacts outside of work, neither of whom developed COVID-19. On July 28, seven days preceding his illness onset, the correctional officer had multiple brief exposures to six IDPs who later tested positive for SARS-CoV-2; available data suggests that at least one of the asymptomatic IDPs transmitted SARS-CoV-2 during these brief encounters. 

Subsequently, VDH and facility staff members reviewed July 28 quarantine unit video surveillance footage and standard correctional officer shift duty responsibilities to approximate the frequency and duration of interactions between the correctional officer and infectious IDPs during the work shift (Table). Although the correctional officer never spent 15 consecutive minutes within 6 feet of an IDP with COVID-19, numerous brief (approximately 1-minute) encounters that cumulatively exceeded 15 minutes did occur. During his 8-hour shift on July 28, the correctional officer was within 6 feet of an infectious IDP an estimated 22 times while the cell door was open, for an estimated 17 total minutes of cumulative exposure. IDPs wore microfiber cloth masks during most interactions with the correctional officer that occurred outside a cell; however, during several encounters in a cell doorway or in the recreation room, IDPs did not wear masks. During all interactions, the correctional officer wore a microfiber cloth mask, gown, and eye protection (goggles). The correctional officer wore gloves during most interactions. The correctional officer’s cumulative exposure time is an informed estimate; additional interactions might have occurred that were missed during this investigation.

**TABLE:** Description, frequency, and duration of close (within 6 ft) interactions between the ill correctional facility employee and six infectious incarcerated or detained persons (IDPs) while their SARS-CoV-2 test results were pending — Vermont, July 28, 2020*

Routine encounter	Description	Typical frequency	Typical duration	Cell door typically open?^†^	Estimated no. of exposures ≤6 ft from infectious persons and cumulative employee July 28 exposure time
**Recreation room use**	Employees open cell doors to allow IDPs to access recreation room one at a time. Observed opportunities for conversation between staff members and IDPs.	Once per 8-hour shift for each IDP	60 seconds	Yes	6 infectious persons x 1 encounter per shift = 6 encounters x 1 minute per encounter = 6 minutes
**Collection of soiled linens and clothes**	Employees collect soiled laundry and provide clean linens and clothing.	Clothes: twice weekly^§^; Linens: once weekly^§^	30–60 seconds	Yes	6 infectious persons x 1 encounter during ill employee’s shift = 6 encounters x 45 seconds = 4.5 minutes
**Showering or recreation**	Employees open doors for IDPs to leave for showering or recreation.	Once daily for each IDP during second shift^¶^	30 seconds	Yes	6 infectious persons x 1 encounter per shift = 6 encounters x 30 seconds = 3 minutes
**Health checks**	Employees conduct health assessments of IDPs.	Once per 8-hour shift for each IDP	60 seconds	During approximately one third of the encounters	6 infectious persons x 1 encounter per shift x 1/3 of encounters with door open = 2 encounters x 1 minute per encounter = 2 minutes
**Medication dispensing**	Employees deliver medication to IDPs. Encounters occur through chutes in doors when possible.	As needed; approximately once per 8-hour shift for each IDP	30–60 seconds	During approximately one third of the encounters	6 infectious persons x 1 encounter per shift x 1/3 of encounters with door open = 2 encounters x 45 seconds per encounter = 1.5 minutes
**Safety checks**	Employees visually check on IDPs through door windows.	Every 15 minutes	<10 seconds	No	None
**Meal delivery and pick-up**	Meals are delivered through food chutes in cell doors; trays are picked up through the same chutes.	Once per 8-hour shift for each IDP	30 seconds	No	None
**Total**	—	—	—	—	**22 encounters; 17 minutes**

**Abbreviation:** COVID-19 = coronavirus disease 2019.

* Standard shift duties and surveillance footage from the quarantine unit were used to characterize routine opportunities for employees and IDPs to have close (within 6 ft) interactions. Observed encounters between the correctional officer and IDPs and typical encounter durations were used to estimate the ill employee’s cumulative exposure time. One correctional staff member is assigned to the quarantine unit per shift and is responsible for performing the tasks described in the table.

^†^ IDPs are not required to wear masks while inside cells. During health checks and medication dispensing interactions when cell doors were open but IDPs remained inside, IDPs did not wear masks despite being within 6 ft of employees without the door as a physical barrier.

^§^ These activities were observed during the course of the correctional officer’s shift because these IDPs were new arrivals to the facility.

^¶^ Surveillance footage was used to estimate the number of encounters between the correctional officer and the six quarantined IDPs pending SARS-CoV-2 test results on July 28.

The correctional officer reported no other known close contact exposures to persons with COVID-19 outside work and no travel outside Vermont during the 14 days preceding illness onset. COVID-19 cumulative incidence in his county of residence and where the correctional facility is located was relatively low at the time of the investigation (20 cases per 100,000 persons), suggesting that his most likely exposures occurred in the correctional facility through multiple brief encounters (not initially considered to meet VDH’s definition of close contact exposure) with IDPs who later received a positive SARS-CoV-2 test result.

Among seven employees with exposures to the infectious IDPs that did meet the VDH close contact definition, one person received a positive test result. Among thirteen employees (including the correctional officer) with exposures to the infectious IDPs that did not meet the VDH close contact definition during contact tracing, only the correctional officer received a positive SARS-CoV-2 test result.

Data are limited to precisely define “close contact”; however, 15 minutes of close exposure is used as an operational definition for contact tracing investigations in many settings. Additional factors to consider when defining close contact include proximity, the duration of exposure, whether the infected person has symptoms, whether the infected person was likely to generate respiratory aerosols, and environmental factors such as adequacy of ventilation and crowding. A primary purpose of contact tracing is to identify persons with higher risk exposures and therefore higher probabilities of developing infection, which can guide decisions on quarantining and work restrictions. Although the initial assessment did not suggest that the officer had close contact exposures, detailed review of video footage identified that the cumulative duration of exposures exceeded 15 minutes. In correctional settings, frequent encounters of ≤6 feet between IDPs and facility staff members are necessary; public health officials should consider transmission-risk implications of cumulative exposure time within such settings. 

## Footnotes

* For the purposes of this report, “IDP” refers to a person held in a prison.

† This activity was reviewed by CDC and was conducted consistent with applicable federal law and CDC policy: 45 C.F.R. part 46, 21 C.F.R. part 56; 42 U.S.C. Sect. 241(d); 5 U.S.C. Sect. 552a; 44 U.S.C. Sect. 3501 et seq.

§ https://www.cdc.gov/coronavirus/2019-ncov/php/public-health-recommendations.html.

¶ https://www.cdc.gov/coronavirus/2019-ncov/php/contact-tracing/contact-tracing-plan/appendix.html#contact.

